# Supplementary material for: Hybrid Models and Biological Model Reduction with PyDSTool
Source: PLoS Comput Biol. 2012 Aug 9;8(8):e1002628. doi: 10.1371/journal.pcbi.1002628 (PMC3415397; doi:10.1371/journal.pcbi.1002628)
Supplement: Text S4 — Complete source code for the PyDSTool package (version 0.88.120504). Includes API documentation and help files linking to web pages. This file is identical to the current public release on Sourceforge.net. (ZIP) [file pcbi.1002628.s004.zip › PyDSTool/html/PyDSTool.Points-module.html]

xml version="1.0" encoding="ascii"?


PyDSTool.Points


| Home | Trees | Indices | Help | PyDSTool |
| --- | --- | --- | --- | --- |

Package PyDSTool ::
Module Points

[hide private]

[frames] | no frames]

# Module Points

source code

```
Point and Pointset enhanced array classes.

(Objects of both classes are mutable.)

    Robert Clewley, February 2006
```


|  |  |
| --- | --- |
| Classes | [hide private] |

VarCaller  
Wrapper for Variable type to call Pointset and return array type.

Point  
N-dimensional point class.

Pointset  
1D parameterized or non-parameterized set of discrete points.

PointInfo  
Structure for storing individual point labels and information
dictionaries within a Pointset object.


|  |  |
| --- | --- |
| Functions | [hide private] |

|  |  |
| --- | --- |
| \_pretty\_print\_label(d)  Internal utility to pretty print point label info. | source code |

|  |  |
| --- | --- |
| comparePointCoords(p1, p2, fussy=False)  Compare two Points, Pointsets, or dictionary of point data, coordinate-wise. | source code |

|  |  |
| --- | --- |
| isparameterized(p)  Returns True if Point or Pointset p is parameterized, False otherwise | source code |

|  |  |
| --- | --- |
| makeNonParameterized(p)  Return a new Pointset stripped of its parameterization. | source code |

|  |  |
| --- | --- |
| pointsToPointset(pointlist, indepvarname=`'``'`, indepvararray=None, indepvartype=<type 'float'>, norm=2)  Generate a Pointset from a list of Point objects (or a singleton Point). | source code |

|  |  |
| --- | --- |
| arrayToPointset(a, vnames=None, ia=None, iname=`'``'`)  Convert an array to a non-parameterized Pointset. | source code |

|  |  |
| --- | --- |
| exportPointset(thepointset, infodict, separator=`'` `'`, precision=12, varvaldir=`'``col``'`, ext=`'``'`, append=False)  Export a pointset to a set of ASCII whitespace- (or user-defined character-) separated data files. | source code |

|  |  |
| --- | --- |
| importPointset(xFileName, t=None, indices=None, sep=`'` `'`, preamblelines=0)  Import ASCII format files containing data points. | source code |

|  |  |
| --- | --- |
| export\_pointset\_to\_CSV(filename, pts)  Simple export that ignores all metadata in pts, including name, tags, norm, etc. | source code |

|  |  |
| --- | --- |
| mergePointsets(pts1, pts2)  Merges two pointsets into a new pointset, preserving (merging) any metadata in each. | source code |

|  |  |
| --- | --- |
| padPointset(pts, pinterval, value\_dict, eps=None)  Pad a pointset pts with values from value\_dict over the interval given by pinterval (pair). | source code |


|  |  |
| --- | --- |
| Variables | [hide private] |

point\_keys = `['coorddict', 'coordarray', 'coordtype', 'norm', ...`

\_1DimplicitSolveMethods = `['newton', 'bisect', 'steffe']`

\_all\_complex = `(<type 'complex'>, <type 'numpy.complexfloating...`

\_all\_float = `(<type 'float'>, <type 'numpy.floating'>, <type '...`

\_all\_int = `(<type 'int'>, <type 'numpy.integer'>, <type 'numpy...`

\_all\_numpy\_complex = `(<type 'numpy.complex128'>, <type 'numpy....`

\_all\_numpy\_float = `(<type 'numpy.float64'>, <type 'numpy.float...`

\_all\_numpy\_int = `(<type 'numpy.int32'>, <type 'numpy.int32'>, ...`

\_complex\_types = `(<type 'complex'>, <type 'numpy.complexfloati...`

\_float\_types = `(<type 'float'>, <type 'numpy.floating'>)`

\_implicitSolveMethods = `['newton', 'bisect', 'steffe', 'fsolve']`

\_int\_types = `(<type 'int'>, <type 'numpy.integer'>)`

\_num\_equivtype = `{<type 'float'>: <type 'numpy.float64'>, <typ...`

\_num\_maxmin = `{<type 'numpy.int32'>: [-2147483648, 2147483647]...`

\_num\_name2equivtypes = `{'float': (<type 'float'>, <type 'numpy...`

\_num\_name2type = `{'float': <type 'numpy.float64'>, 'int': <typ...`

\_num\_type2name = `{<type 'float'>: 'float', <type 'int'>: 'int'...`

\_num\_types = `(<type 'float'>, <type 'int'>, <type 'numpy.float...`

\_pytypefromtype = `{<type 'numpy.int32'>: <type 'int'>, <type '...`

\_real\_types = `(<type 'int'>, <type 'numpy.integer'>, <type 'fl...`

\_seq\_types = `(<type 'list'>, <type 'tuple'>, <type 'numpy.ndar...`


|  |  |
| --- | --- |
| Function Details | [hide private] |

|  |  |
| --- | --- |
| comparePointCoords(p1, p2, fussy=False) | source code |

Compare two Points, Pointsets, or dictionary of point data,
coordinate-wise. If p1 or p2 are Pointsets, their independent variable
values, if present, are \*not\* compared.

fussy option causes point norm order and coordinate types to be
checked too (requires both arguments to be Points or Pointsets).

|  |  |
| --- | --- |
| pointsToPointset(pointlist, indepvarname=`'``'`, indepvararray=None, indepvartype=<type 'float'>, norm=2) | source code |

Generate a Pointset from a list of Point objects (or a singleton
Point).

Include a name for the independent variable if constructing a
parameterized pointset. The independent variable should be a coordinate
of the Points passed, otherwise it can be passed as the optional third
argument.

|  |  |
| --- | --- |
| arrayToPointset(a, vnames=None, ia=None, iname=`'``'`) | source code |

Convert an array to a non-parameterized Pointset. The inclusion of an
optional independent variable array creates a parameterized Pointset.

Coordinate (and independent variable) names are optional: the defaults
are the array indices (and 't' for the independent variable).

|  |  |
| --- | --- |
| exportPointset(thepointset, infodict, separator=`'` `'`, precision=12, varvaldir=`'``col``'`, ext=`'``'`, append=False) | source code |

Export a pointset to a set of ASCII whitespace- (or user-defined
character-) separated data files. Option to list each variable's data in
rows ('across') or in columns ('down'). Existing files of the same names
will be overwritten, unless the 'append' boolean option is set.

NB. If the file extension argument 'ext' is present without a leading
dot, one will be added.

infodict should consist of: keys = filenames, values = tuples of
pointset variable names to export.

|  |  |
| --- | --- |
| importPointset(xFileName, t=None, indices=None, sep=`'` `'`, preamblelines=0) | source code |

```
Import ASCII format files containing data points.
If the first row contains string names then the output
will be a pointset, otherwise a numeric array.

A dictionary is returned, with keys 'vararray' will point to the
data. The 't' argument can specify one of several things:

string: filename to read single-column of time values (same length as
        xFileName)
sequence type: time values (same length as xFileName)
integer: column in xFileName to treat as time data

If used, this leads to and an additional key in the return
dictionary where 't' points to the independent variable array.

Specific columns can be selected for the variable data array by
specifying a list of column indices in argument 'indices'.

The separator used in the ASCII file can be specified by argument
'sep' (defaults to single whitespace character).

preamblelines (positive integer) specifies how many lines to skip before
starting to read data (in case of preceding text) -- default 0.
```

|  |  |
| --- | --- |
| export\_pointset\_to\_CSV(filename, pts) | source code |

Simple export that ignores all metadata in pts, including name, tags,
norm, etc. Data is arranged by row only.

Independent variable is first column, if it exists in pts.

|  |  |
| --- | --- |
| mergePointsets(pts1, pts2) | source code |

Merges two pointsets into a new pointset, preserving (merging) any
metadata in each.

In particular, if each have different accuracy tolerances, the larger
of the two will be used. If each have different 'checklevel' values, the
larger of the two will be used. Point labels will be merged. Names will
also be merged.

If both are parameterized, their independent variable arrays must be
identical. If only one is parameterized, the result will be too. The two
pointsets must be identical in length.

The norm associated with each pointset must be the same.

|  |  |
| --- | --- |
| padPointset(pts, pinterval, value\_dict, eps=None) | source code |

Pad a pointset pts with values from value\_dict over the interval given
by pinterval (pair). For each side of the interval outside of the current
independent variable domain of pts, two new points are added, one at the
outer limit of the interval, and one a distance eps (default the abseps
setting of pts) from the existing closest point in pts.

  


|  |  |
| --- | --- |
| Variables Details | [hide private] |

### point\_keys

Value:
:   ```
    ['coorddict', 'coordarray', 'coordtype', 'norm', 'labels']
    ```

### \_all\_complex

Value:
:   ```
    (<type 'complex'>,
     <type 'numpy.complexfloating'>,
     <type 'numpy.complex128'>,
     <type 'numpy.complex64'>,
     <type 'numpy.complex128'>)
    ```

### \_all\_float

Value:
:   ```
    (<type 'float'>,
     <type 'numpy.floating'>,
     <type 'numpy.float64'>,
     <type 'numpy.float32'>,
     <type 'numpy.float64'>)
    ```

### \_all\_int

Value:
:   ```
    (<type 'int'>,
     <type 'numpy.integer'>,
     <type 'numpy.int32'>,
     <type 'numpy.int32'>,
     <type 'numpy.int8'>,
     <type 'numpy.int16'>,
     <type 'numpy.int32'>,
     <type 'numpy.int64'>)
    ```

### \_all\_numpy\_complex

Value:
:   ```
    (<type 'numpy.complex128'>,
     <type 'numpy.complex64'>,
     <type 'numpy.complex128'>)
    ```

### \_all\_numpy\_float

Value:
:   ```
    (<type 'numpy.float64'>,
     <type 'numpy.float32'>,
     <type 'numpy.float64'>)
    ```

### \_all\_numpy\_int

Value:
:   ```
    (<type 'numpy.int32'>,
     <type 'numpy.int32'>,
     <type 'numpy.int8'>,
     <type 'numpy.int16'>,
     <type 'numpy.int32'>,
     <type 'numpy.int64'>)
    ```

### \_complex\_types

Value:
:   ```
    (<type 'complex'>, <type 'numpy.complexfloating'>)
    ```

### \_num\_equivtype

Value:
:   ```
    {<type 'float'>: <type 'numpy.float64'>,
     <type 'int'>: <type 'numpy.int32'>,
     <type 'numpy.integer'>: <type 'numpy.int32'>,
     <type 'numpy.floating'>: <type 'numpy.float64'>,
     <type 'numpy.int8'>: <type 'numpy.int32'>,
     <type 'numpy.int16'>: <type 'numpy.int32'>,
     <type 'numpy.int32'>: <type 'numpy.int32'>,
     <type 'numpy.int32'>: <type 'numpy.int32'>,
    ...
    ```

### \_num\_maxmin

Value:
:   ```
    {<type 'numpy.int32'>: [-2147483648, 2147483647],
     <type 'numpy.float64'>: [-inf, inf]}
    ```

### \_num\_name2equivtypes

Value:
:   ```
    {'float': (<type 'float'>,
               <type 'numpy.floating'>,
               <type 'numpy.float64'>,
               <type 'numpy.float32'>,
               <type 'numpy.float64'>),
     'int': (<type 'int'>,
             <type 'numpy.integer'>,
             <type 'numpy.int32'>,
    ...
    ```

### \_num\_name2type

Value:
:   ```
    {'float': <type 'numpy.float64'>, 'int': <type 'numpy.int32'>}
    ```

### \_num\_type2name

Value:
:   ```
    {<type 'float'>: 'float',
     <type 'int'>: 'int',
     <type 'numpy.integer'>: 'int',
     <type 'numpy.floating'>: 'float',
     <type 'numpy.int8'>: 'int',
     <type 'numpy.int16'>: 'int',
     <type 'numpy.int32'>: 'int',
     <type 'numpy.int32'>: 'int',
    ...
    ```

### \_num\_types

Value:
:   ```
    (<type 'float'>,
     <type 'int'>,
     <type 'numpy.floating'>,
     <type 'numpy.integer'>)
    ```

### \_pytypefromtype

Value:
:   ```
    {<type 'numpy.int32'>: <type 'int'>,
     <type 'numpy.float64'>: <type 'float'>}
    ```

### \_real\_types

Value:
:   ```
    (<type 'int'>,
     <type 'numpy.integer'>,
     <type 'float'>,
     <type 'numpy.floating'>)
    ```

### \_seq\_types

Value:
:   ```
    (<type 'list'>, <type 'tuple'>, <type 'numpy.ndarray'>)
    ```

  


| Home | Trees | Indices | Help | PyDSTool |
| --- | --- | --- | --- | --- |

|  |  |
| --- | --- |
| Generated by Epydoc 3.0.1 on Fri May 4 15:24:03 2012 | http://epydoc.sourceforge.net |
